# The Physical Activity, Imaging, and Ambulatory Testing (PHIAT) Project: Protocol for a High-Frequency Ambulatory Assessment Study

**DOI:** 10.2196/66290

**Published:** 2025-10-09

**Authors:** Jonathan G Hakun, Daniel B Elbich, Jessie N Alwerdt, Ashley M Tate, Jennifer L Coyl, Bethany M Kanski, Tian Qiu

**Affiliations:** 1 Department of Neurology, Pennsylvania State University, Penn Sate Milton S. Hershey Medical Center, 500 University Drive, H037, Hershey, PA, 17033, United States, 1 7175310003 ext 287082; 2 Department of Psychology, Pennsylvania State University, University Park, PA, United States; 3 Department of Public Health Sciences, Pennsylvania State University, Hershey, PA, United States; 4 Center for Healthy Aging, Pennsylvania State University, University Park, PA, United States

**Keywords:** self-regulation, executive control, EMA, ambulatory cognitive assessment, physical activity, imaging, ambulatory testing, ambulatory assessment, emotion, exercise, self-monitor, health-promoting behavior

## Abstract

**Background:**

Multiple independent lines of research on self-regulation point to executive cognitive ability (executive function/cognitive control) as a factor that underlies the capacity to successfully regulate one’s thoughts, emotions, and behavior. Critically, while leading theories point to the role of executive control in modifying “in-the-moment” regulatory processes (eg, enacting physical activity, resisting a poor dietary choice, reflecting on a stressful experience), few studies have tested the executive hypothesis at this timescale. Moreover, given the normative changes in executive control across the adult lifespan, it is essential to understand how cognitive aging might impact these processes.

**Objective:**

The Physical Activity, Imaging, and Ambulatory Testing (PHIAT) project was designed to test how variation in executive control at multiple timescales (from moment-to-moment within days to age differences across decades) influences self-regulation across the adult lifespan.

**Methods:**

A 14-day, high-frequency, ambulatory assessment protocol was designed for this study. The study was conducted in a measurement burst design and included a 14-day ecological momentary assessment (EMA) protocol involving 6 assessments per day. Ultra-brief ambulatory cognitive assessments of multiple domains of cognition were included in the EMA protocol. Throughout the measurement burst, participants also wore 3 activity monitors on the hip, thigh, and wrist to measure physical activity/exercise, measure sedentary behavior, and self-monitor physical activity behavior, respectively.

**Results:**

A total of 221 participants ranging from 18 to 89 years of age completed the PHIAT protocol over the course of 2021-2024, and data collection is complete. EMA data were collected from participants reflecting a wide range of psychosocial factors surrounding participation in health-promoting behaviors (motivation, intention, stress, built environment, and social cognitive factors). This EMA data stream is complemented by data from high-frequency, ambulatory cognitive assessments measuring processing speed, working memory capacity, inhibitory control, and divided/sustained attention administered 5 times per day throughout the 14-day burst. In addition, health-promoting behaviors, including sleep, diet, hydration, physical activity, and exercise, were assessed throughout the 14-day burst through a combination of EMA self-report and continuous activity monitoring.

**Conclusions:**

A rich, high-frequency dataset was generated by the PHIAT project that will provide a range of novel insights into the motivational factors, information processing, and environmental factors that surround self-regulation of health-promoting behavior.

## Introduction

### Background

A defining feature of human cognition is the ability to behave in a goal-directed manner in the face of a myriad of competing, and often salient, alternatives (our ability to self-regulate) [1-3]. Behaviors that promote long-term health, such as physical activity (PA), are often described as “effortful” or “difficult” and thus require effective self-regulatory control to align momentary behavior (going for a walk) with long-term goals (to remain healthy) [4-8]. Regular PA is associated with a wide range of long-term health benefits, including reduced risk for preventable chronic disease (eg, cardiovascular disease and type 2 diabetes) and age-related neuropathology (eg, Alzheimer disease and related dementias) [9-14]. In addition, regular PA may support long-term cognitive health throughout the adult lifespan [15,16]. Thus, a virtuous cycle has been speculated to exist between cognition and PA behavior [5,17,18]. Critically, however, substantial individual differences exist in the ability to translate one’s motivation and goals to maintain or increase PA into overt daily behavior (known as the PA “intention-behavior gap”) [6,8,19,20]. Despite great efforts to understand why such a gap exists and how to successfully intervene, the antecedents and moderators of successful self-regulation remain poorly understood. Moreover, it remains unclear whether these factors play a stable role in PA self-regulation across the adult lifespan.

Recent attention has turned to cognitive ability, specifically executive control, as a possible source of individual differences in self-regulatory capacity [21-24]. Several recent studies evaluating the executive hypothesis, including our own work, support this claim showing that control over information processing moderates and independently contributes to health outcomes and health promotion behavior over and above self-reported intentions or tendency to self-regulate [6,21,22,25,26]. However, executive control abilities are not stationary and may vary as a function of time (eg, time of day) and context (eg, in response to a stressful interaction) [27-29]. Moreover, the underlying neurocognitive resources (the frontoparietal brain structure and function) are particularly subject to age-related decline and alteration [30-38]. These multitimescale alterations may influence *when* an individual is prepared to successfully self-regulate and may modify the process of self-regulation over the course of the adult lifespan. However, few studies have examined these mechanisms across these timescales or in an age-diverse sample.

### The Project

The Physical Activity, Imaging, and Ambulatory Testing (PHIAT) project was a high-frequency, ambulatory assessment study conducted over the course of 2019-2024. The project was sponsored by the National Institute on Aging of the National Institutes of Health as part of an R00 Career Development Award (R00AG056670; principal investigator: JGH). The project was designed as an adult lifespan, microlongitudinal observational study of real-time variation in cognitive executive control and self-regulation of health-promoting behavior. The overall aims of the PHIAT project were as follows: (1) to examine variation in executive control and self-regulation of PA in real time and (2) to examine age-variant and invariant contributions of brain structure, function, and executive control in self-regulation of health-promoting behaviors. The overarching hypothesis examined by the PHIAT project was that *executive control is a resource upon which successful self-regulation depends* (an *executive hypothesis* of self-regulation; see also [8,22-24]).

To accomplish the PHIAT project aims, we chose an ecological momentary assessment (EMA) approach. The EMA protocol was conducted over a 14-day measurement burst (6 assessments/d). This approach was complemented by the contemporaneous use of multiple wearable activity monitors (to measure PA, sedentary behavior, and self-monitoring behavior) and the administration of ultra-brief, ambulatory cognitive assessments during EMA surveys to examine short-term variation (within- and between-day) in multiple domains of cognition.

These high-frequency data streams were designed to be integrated at multiple timescales to accomplish the study aims. A central focus of the project was on short-term variation in executive control. In a previous set of studies, we rigorously designed and parameterized ultra-brief adaptations of complex span working memory tasks for EMA administration [27]. Based on our development and testing, we selected the ultra-brief (~1 min) rotation span task as our primary indicator of executive control in PHIAT. We also included other ultra-brief experimental indices of attention and executive control, including a go/no-go task and a multiple object tracking task, as well as a previously validated indicator of processing speed (symbol search task) [39]. Each of these tasks was administered during 5 of the 6 daily EMA surveys (morning survey, plus 4 beeped surveys), providing a detailed time series of variation in multiple domains of cognition both within and between days in the study.

### Conceptual Framework

#### Overview

In many studies, successful self-regulation is inferred from the exhibition of a target behavior (eg, meeting recommended levels of weekly PA, achieving a healthy diet, adaptive affective responses, and successful behavior change). In the PHIAT project, we aimed to test the executive hypothesis at a more granular level by extending two existing frameworks to a higher-frequency sampling approach: a *dynamic action control framework* and a *momentary contextual reactivity framework*. A major assumption underlying this approach is that no one is *always* successful at regulating their thoughts, emotions, and behavior, and that by intensively monitoring behavior, we might be able to generate a more detailed picture of the factors that drive self-regulatory success and failure (ie, moderators).

#### Dynamic Action Control Framework

**Figure 1.**
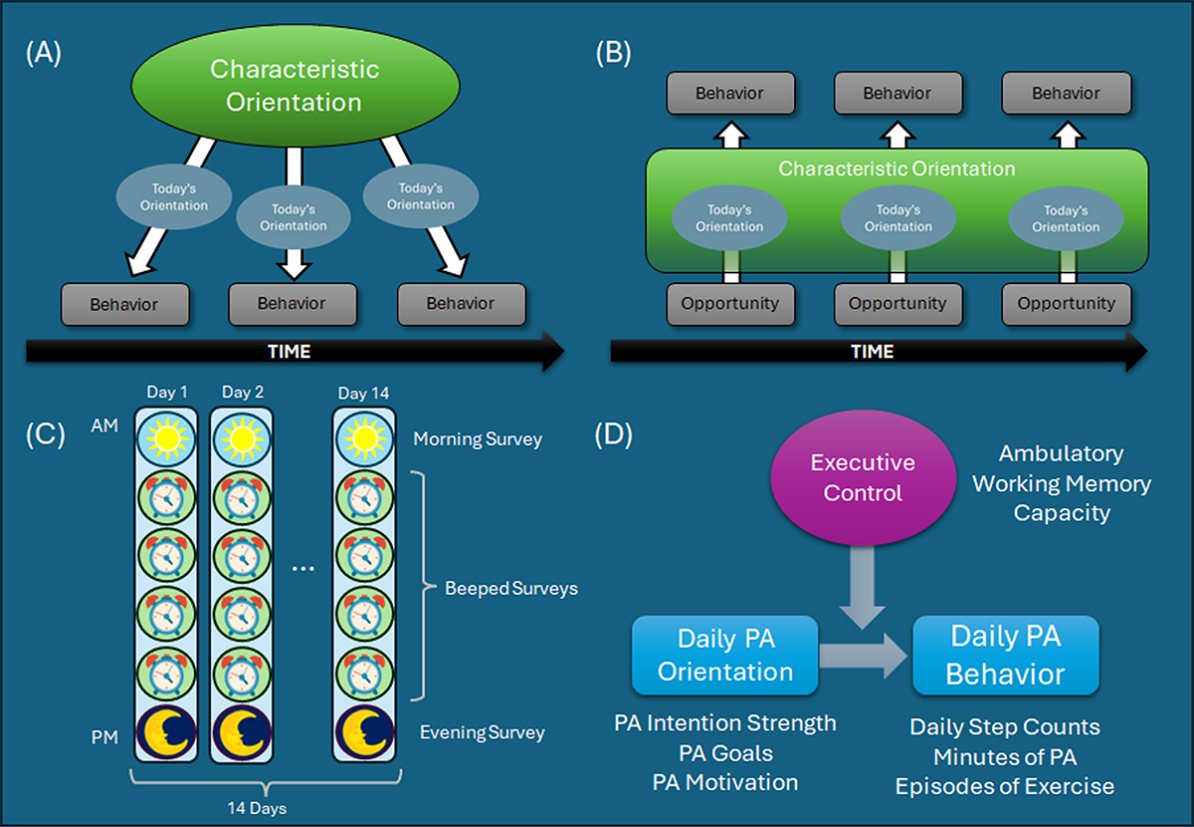
The Physical Activity, Imaging, and Ambulatory Testing (PHIAT) project conceptual framework. Two frameworks for operationalizing self-regulation as a within-person process are included in the PHIAT project. (A) *Dynamic action control framework*: The ecological momentary assessment (EMA) protocol is designed to examine whether a person’s daily orientation toward PA (motivation, intentions, and goals) manifests in that day’s PA behavior. (B) *Momentary contextual reactivity framework*: The EMA protocol surveils for PA opportunities and examines whether a person’s behavior in those contexts is consistent with their characteristic or daily PA orientation. (C) The EMA protocol lasted for 14 days and included 6 assessments per day. (D) General within-person analytical approach. Multilevel moderation analyses are planned to examine the executive hypothesis, which predicts a stronger association between today’s intention to be physically active and today’s physical activity behavior on days with higher working memory capacity. PA: physical activity.

The process of translating/realizing one’s motivation into real-world behavior has been a major focus of study for decades [1,20,40]. Among research focused on the psychological antecedents of behavior, the formation of an intention has emerged as among the most proximal to action [20,41]. The action control framework proposes that there may be several factors that guide the translation of (or failure to translate) intentions into behavior. In the case of PA, modifiers of action control ranging from psychological (eg, social cognitive variables, personality, affect, and identity) to behavioral (eg, habit/previous behavior) have been well-studied [41]. A limitation of previous work has been the focus on explaining the PA intention-behavior gap in cross-section (ie, *who* successfully translates their intentions into behavior). In the PHIAT project, we extended this framework to accommodate day-to-day variation in factors that might help explain the gap at a more process-oriented level (ie, *when* do individuals successfully translate their intentions into behavior; see also [42]; Figure 1A). Several aspects of PA *orientation* (intention, goals, perceived control, and social cognitive factors) were monitored on a daily basis throughout the protocol, with a focus on short-term variation in executive control as the primary hypothesized modifier. In addition, the inclusion of an adult lifespan sample provides the opportunity to evaluate how age-associated changes in factors surrounding action control modify the process.

#### Momentary Contextual Reactivity Framework

The second framework we extended to examine self-regulation in daily life is the contextual reactivity approach. Contextual reactivity refers to an individual’s response to an exposure in their everyday environment. In previous research, this approach was used to study other domains of self-regulation (eg, affective reactions to everyday stressors) [43,44], and reactivity under these circumstances is often taken to reflect a stable trait characteristic even if sampled with a higher-frequency approach (eg, daily diary). In our recent work, we found that short-term variation in executive control (momentary working memory capacity) may modify a person’s reaction to an everyday stressor exposure, suggesting that the momentary *state* of one’s executive control ability may play a role in determining their reaction [28]. In PHIAT, we aimed to, similarly, capture how individuals react when exposed to an environment that is conducive/supportive of enacting PA behavior and examine whether that reaction is consistent with an individual’s overall orientation for PA or even their orientation for that day. PA contextual reactivity was indicated by behavior during exposure to environments that were conducive to PA (PA *opportunities*; Figure 1B). PA opportunities were assessed during all beeped and evening EMA surveys.

In each case, by closely monitoring motivational and contextual dynamics, the PHIAT protocol represents a high-frequency, natural experimental framework and provides a stronger test of the executive hypothesis than can be generated through cross-sectional, observational approaches. Our central analytical approach involves examining whether associations between PA orientation, PA opportunities, and PA behavior are moderated by within- and between-person variation in executive control (eg, rotation span performance either during that moment/day or average performance across all observations; Figure 1D).

Another key feature of the PHIAT project was the integration of a commercially available wrist-worn activity monitor and the software used to administer the EMA protocol. The face of the activity monitor was configured to only display the time of day, while information about accumulated activity (step counts) was transmitted via Bluetooth to the EMA software installed on investigator-provided smartphones. Participants were provided the opportunity to check their step counts throughout the day using a *check my steps* button made available through the EMA software main menu. Self-monitoring behavior, a construct central to many theories of self-regulation [45], was operationalized as the number of times a participant accessed the *check my steps* button, providing an objective measure of real-time self-monitoring behavior.

## Methods

### Participants

Participants were recruited from a combination of social media postings, internal institutional resources (eg, “StudyFinder”), secondary recruitment from other studies conducted at Penn State, and the community surrounding health care sites of Penn State Health system. Participants were included if they were aged 18 years and older, were ambulatory, were fluent in English, had access to a reliable cellular network or Wi-Fi connectivity for onboarding, and had an email account for communication of remote consent procedures. Participants were excluded if they had a motor or visual impairment that would interfere with the operation of the study-provided smartphone, had a history of neurological injury or disease or significant health problem that could be complicated by PA, were unable to consent, were younger than 18 years, or were a prisoner.

### Ethical Considerations

All participants provided written informed consent and received compensation for their participation. The compensation rate for the study was US $5 per day of participation (14 d × US $5 = US $70), with a US $30 incentive payment for completing >75% of expected surveys (maximum compensation: US $100).

All experimental procedures were approved by Pennsylvania State University’s Institutional Review Board for the ethical treatment of human participants under protocol number STUDY00013106. All participants provided electronic informed consent via REDCap prior to engaging with any study procedures. All study data were deidentified to ensure participant privacy and confidentiality.

### Materials and Procedure

The study protocol involved 3 phases: (1) participants completed a remote study onboarding visit where informed consent was documented, a telephone adaptation of the Montreal Cognitive Assessment [46] was administered, and participants received a study overview, study materials (study-provided smartphone, tablet, activity monitoring wearables, and EMA app), and instructions for completing the EMA surveys and ambulatory cognitive assessments; (2) questionnaires were administered on a study-provided tablet during the week leading up to the EMA measurement burst; and (3) participants completed a 14-day EMA measurement burst while wearing the activity monitors.

### EMA Protocol

Participants were asked to carry a study-provided smartphone for a period of 14 days (Xiaomi Mi A2 model phones configured with Android One OS, v8.0; Xiaomi Corporation, Beijing, China). A custom Android EMA mobile app, the “Mobile Monitoring of Cognitive Change” (M2C2) app, was loaded onto the study-provided phones and used to administer the EMA protocol, including self-report survey items and 4 ambulatory cognitive assessments. Phones were configured into a “kiosk” mode where only the study app was available to the participant. EMA surveys were administered to each participant over 14 consecutive days in a measurement burst design. Each day involved 1 self-initiated *morning* survey, followed by 4 pseudorandomly signaled *beeped* surveys, and 1 self-initiated *evening* survey (Figure 1C). Participants were instructed to begin the morning survey as soon as they woke up and the evening survey before going to bed. At the start of the study, participants provided their typical waking time. From this information, the EMA protocol was initialized so that the first beeped survey would be delivered at a pseudorandomly determined time within 2 hours of their reported waking time. The remaining 3 beeped surveys were each separated by 3.75 hours and with a start time that was pseudorandomly jittered each day in the burst by up to 30 minutes. Beeped surveys “timed-out” (were unavailable) if the participant did not respond within 30 minutes of the notification. All ambulatory cognitive assessments were administered at the end of each morning and beeped survey but not during the evening survey to limit participant burden.

### EMA Measures

#### Ambulatory Cognitive Assessments

**Figure 2.**
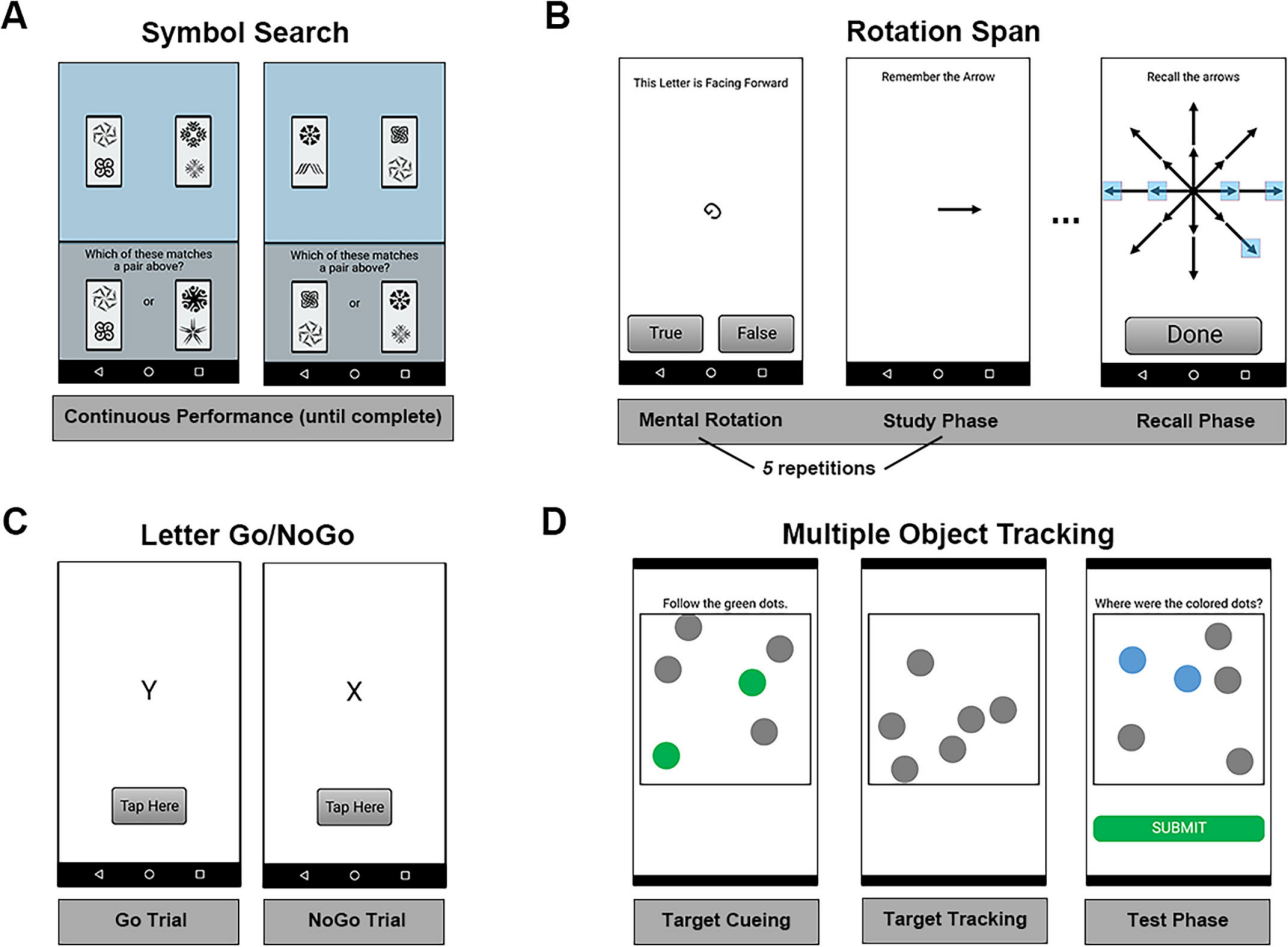
Ambulatory cognitive assessments. (A) symbol search task, (B) rotation span task, (C) letter go/no-go, and (D) multiple object tracking. Each was administered 5 times per day throughout the 14-day protocol.

Four ambulatory cognitive assessments from the M2C2 platform were administered during morning and beeped surveys (Figure 2). A brief description of each task and chosen parameters is provided below, and a complete and detailed description of each task can be found elsewhere [47].

##### Symbol Search

The symbol search task was selected as a measure of processing speed. Eighteen trials were administered during each session. Three target tiles were presented during each trial, and lure tiles occurred on 50% of trials.

##### Rotation Span

The rotation span task was selected as a measure of working memory capacity. Three trials were administered during each session. The set size was 5 for each trial.

##### Letter Go/NoGo

The letter go/no-go task was selected as a measure of response inhibition. A total of 24 trials were administered during each session. “X” was the “NoGo” target, and all other letters of the English alphabet were used as possible “Go” stimuli. The go/no-go ratio was 5:1.

##### Multiple Object Tracking

The multiple object tracking task was selected as a measure of divided attention. Six trials were administered during each session, including 3 trials where the dots moved at a slower speed and 3 trials where the dots moved at a faster speed. The sequence of trials was fixed so that the 3 slow trials were administered first, followed by the 3 fast trials.

### EMA Surveys

Brief ~1-minute self-report surveys with items covering the domains listed below were administered at the beginning of each survey administration (ie, *session*). EMA surveys were designed to provide an in-depth description of participants’ daily experiences, exposures, thoughts, and behaviors (all surveys), as well as their outlook and expectations for each day (during the morning surveys) and review and inventory of each day as a whole (during the evening surveys). A particular emphasis was placed on participation, and factors surrounding participation, in healthful behaviors (PA being of primary interest). For example, each morning survey included items such as “Today, I intend to be *physically active*.” These items were included to model daily within-person self-regulatory processes, including associations between daily variation in PA motivation and behavior (measured via activity monitors, see below). Throughout each day, exposures that might facilitate or act as a barrier to engagement in healthful behaviors (eg, opportunities for PA, exposure to stressful events) were sampled to model short-timescale variation in engagement and other processes (eg, moment-to-moment, within-day variation in PA behavior). Table 1 presents a complete list of all items included in the protocol.

**Table 1.** Physical Activity, Imaging, and Ambulatory Testing (PHIAT) ecological momentary assessment protocol.

Item	Survey	Type	Response options
Context			
Location	AM^a^, BEEP^b^, PM^c^	Multi	Multiple options
Company	AM, BEEP, PM	Multi	Multiple options
Healthy behaviors			
Physically active	AM, BEEP, PM	Radio	Yes/no
PA^d^ intensity^e^	AM, BEEP, PM	Radio	Light, moderate, vigorous
PA duration^e^	AM, BEEP, PM	Select	Minutes (5-min increments)
PA exercise^e^	AM, BEEP, PM	Radio	Yes/no
PA environment	BEEP, PM	Slider	Not at all-extremely
Food	AM, BEEP, PM	Radio	Yes/no
Food healthy rating^f^	AM, BEEP, PM	Slider	Not at all-extremely
Water	AM, BEEP, PM	Radio	Yes/no
Ounces consumed^g^	AM, BEEP, PM	Select	0‐32 (4-oz increments)
Other consumed items	AM, BEEP, PM	Multi	Multiple options
Sleep			
Refreshing	AM	Slider	Not at all-extremely
Disruptions	AM	Slider	Not at all-extremely
Difficulty onset	AM	Slider	Not at all-extremely
Time in bed	AM	Time	Hour/min
Time waking	AM	Time	Hour/min
Affect			
Arousal	AM, BEEP, PM	Slider	Sleepy/tired-awake/alert
Mood	AM, BEEP, PM	Slider	Bad-good
Content	AM, BEEP, PM	Slider	Not at all-extremely
Worry	AM, BEEP, PM	Slider	Not at all-extremely
Happy	AM, BEEP, PM	Slider	Not at all-extremely
Fatigued	AM, BEEP, PM	Slider	Not at all-extremely
Stressed	AM, BEEP, PM	Slider	Not at all-extremely
Sad	AM, BEEP, PM	Slider	Not at all-extremely
Other			
Pain	AM, BEEP, PM	Slider	Not at all-extremely
Subjective age	AM, PM	Slider	0‐100
Forecasting			
Expect today stressful	AM	Slider	Not at all-extremely
Expect today busy	AM	Slider	Not at all-extremely
Expect today distractions	AM	Slider	Not at all-extremely
Expect today enjoyable	AM	Slider	Not at all-extremely
Expect today social	AM	Slider	Not at all-extremely
Intentions			
PA intention	AM	Slider	Not at all-extremely
Healthy meals intention	AM	Slider	Not at all-extremely
Hydrated intention	AM	Slider	Not at all-extremely
Sleep intention	AM	Slider	Not at all-extremely
PA goal			
Step count goal	AM	Radio	Yes/no
Step goal amount^h^	AM	Select	0‐20000 (500-step increments)
Goal perceived control^h^	AM	Slider	Not at all-extremely
Goal efficacy^h^	AM	Slider	Not at all-extremely
Goal cog expectations^h^	AM	Slider	Not at all-extremely
Goal affect expectations^h^	AM	Slider	Not at all-extremely
Goal utility^h^	AM	Slider	Not at all-extremely
Goal set	PM	Radio	Yes/no
Goal achieved^i^	PM	Radio	Yes/no
Goal realistic^i^	PM	Slider	Not at all-extremely
Goal easy^i^	PM	Slider	Not at all-extremely
Goal rewarding	PM	Slider	Not at all-extremely
Goal within control^i^	PM	Slider	Not at all-extremely
Goal facilitators^i^	PM	Multi	Multiple options
Goal attribution^i^	PM	Slider	Not at all-extremely
Goal barriers^i^	PM	Multi	Multiple options
Subjective cognition			
Slow	BEEP, PM	Slider	Not at all-extremely
Foggy	BEEP, PM	Slider	Not at all-extremely
Sharp	BEEP, PM	Slider	Not at all-extremely
Concentration	BEEP, PM	Slider	Not at all-extremely
Everyday memory failures	PM	Multi	Multiple options
Everyday attention failures	PM	Multi	Multiple options
Thoughts and disruptions			
Distractions	BEEP, PM	Slider	Not at all-extremely
Busy	BEEP, PM	Slider	Not at all-extremely
Control train of thought	BEEP, PM	Slider	Not at all-extremely
Mind-wandering	BEEP, PM	Slider	Not at all-extremely
Forgetting	BEEP, PM	Slider	Not at all-extremely
Stress			
Stressful exposure	BEEP, PM	Radio	Yes/no
Impact of stressors^j^	BEEP, PM	Slider	Not at all-extremely
Stress avoidance^j^	BEEP, PM	Slider	Not at all-extremely
Daily wrap-up			
Good day	PM	Slider	Not at all-extremely
Satisfied with activities	PM	Slider	Not at all-extremely
Satisfied with social life	PM	Slider	Not at all-extremely
Sense of belonging	PM	Slider	Not at all-extremely
Responsibility impact	PM	Slider	Not at all-extremely
Memory interference	PM	Slider	Not at all-extremely
Memory appraisal	PM	Slider	Not at all-extremely
Attention interference	PM	Slider	Not at all-extremely
Attention appraisal	PM	Slider	Not at all-extremely
Overall PA today	PM	Slider	Not at all-extremely
Overall PA effort	PM	Slider	Not at all-extremely
Overall PA satisfaction	PM	Slider	Not at all-extremely
Exercise today	PM	Radio	Yes/no
Exercise enjoyment^k^	PM	Slider	Not at all-extremely
Exercise appraisal^k^	PM	Slider	Not at all-extremely
Overall food healthy	PM	Slider	Not at all-extremely
Overall food effort	PM	Slider	Not at all-extremely
Overall food satisfaction	PM	Slider	Not at all-extremely
Overall food enjoyment	PM	Slider	Not at all-extremely
Overall hydration effort	PM	Slider	Not at all-extremely
Overall hydration satisfaction	PM	Slider	Not at all-extremely

a AM: morning survey.

b BEEP: beeped survey.

c PM: evening survey.

d PA: physical activity.

e Branched items under parent branching item “Physically active.”

f Branched items under parent branching item “Food.”

g Branched items under parent branching item “Water.”

h Branched items under parent branching item “Step count goal.”

i Branched items under parent branching item “Goal set.”

j Branched items under parent branching item “Stressful exposure.”

k Branched items under parent branching item “Exercise today.”

### PA Monitoring

#### ActiGraph

Participants were asked to wear an ActiGraph activity monitor (Model GT3X, ActiGraph LLC, Pensacola, FL) at their hip (on their belt or waistband) from waking until bedtime throughout the 14-day measurement burst. ActiGraph GT3Xs were programmed to sample at 30 Hz, and participants were also provided a belt clip to secure the device.

#### ActivPAL

Participants were asked to wear an ActivPAL activity monitor (Model 4, PAL Technologies, Ltd, Glasgow, Scotland) affixed to their nondominant thigh via Hypafix (BSN Medical, Hull, United Kingdom) 24 hours per day throughout the 14-day measurement period. The device was programmed to sample at 20 Hz and was provided to participants wrapped in a nitrile sleeve and hypoallergenic athletic tape.

#### Commercial Activity Monitor

A wrist-worn commercial activity monitor was provided to participants for continuous tracking of step counts. The screen of this device was programmed to only display the time (clock) and battery charge level. Data from this device were transmitted via Bluetooth to the EMA app for self-monitoring of PA. Self-monitoring behavior was operationalized as the number of times participants checked their step counts via the EMA app on the provided smartphone.

### Participant Background and Characterization

#### Demographics

A demographics questionnaire was included that captured information on participants’ backgrounds, including age, race, ethnicity, education level, occupation status, income, living situation (living alone/with other individuals), and caregiver status.

#### PA and Social Cognitive Factors

Self-reported PA and motivational factors were assessed via the International Physical Activity Questionnaire [48], Outcome Expectations for Exercise [49], Self-Efficacy for Exercise [50], and the Self-Report Habit Index (adapted to capture habit strength for PA [51]).

#### Self-Regulation

Indicators of a trait orientation toward self-regulation included the Consideration of Future Consequences Scale [52], the 10-item Personality Inventory [53], the Self-Control Scale [54], and the Emotion Regulation Questionnaire [55].

#### Social Connectedness and Well-Being

Three indicators of social connectedness (social network, isolation, and loneliness) were collected, including the Oslo Social Support Scale [56], the NSHAP Social Disconnectedness Scale [57], and the UCLA 3-item Loneliness Scale [58]. Well-being was assessed via the Satisfaction With Life Scale [59], Subjective Happiness Survey [60], Mindful Attention Awareness Scale [61], Food Insecurity Module [62], Self-Rated Health [63], Perceived Vulnerability to Illness [64], Recent Colds History [64], and the PROMIS Physical Function Short Form [65].

#### Aging Attitudes

Two questionnaires assessing attitudes toward aging were collected, including the Awareness of Age-Related Change [66] and Expectations Regarding Aging [67] scales.

### Statistical Analysis Approach

The high-frequency sampling methodology inherent to EMA protocols results in complex nested data structures. Each observation (“moment”) can be considered as nested within a day of the protocol and, in turn, nested within an individual. Statistical analysis approaches that can adequately account for these complex data structures will be used to achieve the study aims. Mixed-effects modeling and dynamic structural equation modeling are two leading approaches to analyzing EMA data that will be used [68]. In each case, all observations will be “centered” in a manner that provides for unambiguous interpretation of effects at each “level” of the study design (ie, within-person, between-person) [69].

For analyses related to the *dynamic action control* framework, linear mixed-effects models will be estimated where day-to-day variation in behavior (eg, daily step counts) will be regressed on within- and between-person (ie, “characteristic”) variation in morning intention ratings. To examine how the characteristic level of, for example, PA intention strength modifies associations between daily variation in PA intention strength and daily PA behavior, cross-level interactions between characteristic PA intentions, and within-person daily variation in PA intention strength will be included in the model. For analysis of *momentary contextual reactivity* effects, linear mixed-effects models will be estimated to examine how momentary variation in the outcome of interest (eg, negative affect, PA behavior between surveys) is affected by exposure to a given context (eg, stressor, opportunity for PA), consistent with our previous work [28,70].

In each case (dynamic action control and momentary contextual reactivity), the central hypothesis being tested in the PHIAT project, the *executive hypothesis* of self-regulation, will be examined at both the between- and within-person levels. To test the hypothesis, within-person variation and characteristic level of executive control (momentary and average performance on the ambulatory cognitive assessments, respectively) will be included as moderators of action control and contextual reactivity effects. For example, we will include within and between interactions between momentary and characteristic working memory capacity (rotation span task performance) and PA intention strength in dynamic action control models to evaluate how within- and between-person executive control ability modifies associations between intentions to engage in PA and PA behavior.

## Results

The aims of this study were revised in response to the COVID-19 pandemic, as the opportunity to conduct in-person magnetic resonance imaging (MRI) with higher risk populations (ie, adults ≥60 y of age) was lost for the majority of the project period. The entire study was pivoted to a remote format in response to the pandemic, including all study procedures (eg, electronic consent, video conference–based onboardings, sanitization, and courier-based delivery and return of all study devices), and all study data were collected after the onset of the pandemic. The sample size for the EMA protocol was increased as resources were redirected from MRI scans to additional recruitment.

We proposed to recruit 150 participants for the PHIAT project. After pivoting the study to fully remote procedures and closing the MRI study, we revised the proposed EMA protocol sample size to 250. A total of 302 participants were screened for eligibility, 243 participants signed informed consent, and 22 withdrew from the study. A total final sample of 221 participants, ranging from 18 to 89 years of age, completed the EMA protocol (mean age = 56.7, SD = 17.9 years). Participants in the final sample were 80% female. About 86% of the sample identified as White, 1.4% Black or African American, 4.5% Asian, 0% Native Hawaiian or other Pacific Islander, 0% American Indian or Alaska Native, 3.2% reported more than one race, and 1.4% identified as Hispanic or Latino. Recruitment began in June 2021 and continued through May 2024 when the study was completed.

## Discussion

### Principal Findings

We expect data generated by the PHIAT project to provide new insights into the intersection of cognitive executive control ability, self-regulation, and health-promoting behaviors. In particular, we anticipate that the ability to link the high-frequency assessment protocol and activity monitoring devices at multiple timescales will help bring the dynamic processes surrounding self-regulatory success and failure into sharper relief. Few previous studies have examined how short-timescale variation in these factors interacts in determining participation in health-promoting behavior, and the current study is an initial step in moving from a cross-sectional/individual difference approach to action control, contextual reactivity, and the executive hypothesis toward a within-person, process-level description.

The 3 activity monitoring devices selected for the study, the thigh-worn ActivPAL monitors, hip-worn ActiGraph GT3X devices, and the wrist-worn commercial fitness monitor, were each chosen to capture different aspects of PA and self-regulatory behavior. The long battery-life ActivPAL devices, being affixed to the thigh, allowed for near-continuous measurement of posture and movement throughout the 14-day protocol. From the ActivPAL devices, we expect to obtain high-fidelity estimates of total PA volume (eg, step counts) as well as estimates of time spent in various postures/behaviors (eg, stepping time, sitting time). The ActiGraph GT3X devices are a widely used tool for estimating PA at different intensity thresholds (eg, moderate-to-vigorous intensity PA) [71]. Because the sample includes adults throughout the lifespan, the selection of activity intensity cut points will require careful consideration [72]. Finally, while the activity data generated by the wrist-worn fitness watch is not expected to be an outcome of interest, participants’ use and interaction with the “check my steps” feature of the EMA application (which was connected via Bluetooth to this device) will be examined in studies of how self-monitoring of PA behavior is influenced by goal setting and variation in executive control.

The wide age range of the PHIAT study sample will allow for investigation into how age-associated changes in each factor either independently or mutually alter self-regulatory processing throughout the adult lifespan. Previous work has shown that many of the individual factors under study undergo normative age-associated change. For example, it is well-established that older age is associated with reductions in PA, increases in sedentary behavior, and reduced executive cognitive ability [73-79]. In addition, previous work on psychosocial aspects of aging (eg, affective and cognitive well-being) has demonstrated that factors such as perspective, world view, and cognitive capacity may play a role in altering the strategies we select and rely on to manage our thoughts, emotions, and behavior in older adulthood [24,80-82]. The PHIAT project offers an opportunity to connect this line of work from the fields of psychosocial aging and human development to these areas of health promotion theory.

### Limitations

While the strengths of the PHIAT project study design reviewed above offer the potential to gain new insights into self-regulation of health promotion, there are several limitations. While every effort was made to recruit a generalizable and representative sample, the final sample was disproportionately female and White. We suspect this was driven, in part, by our advertising and recruiting methods, which included a health system–associated marketing campaign over social media. In addition, despite recruiting community members throughout the region with our sampling methodology, the PHIAT project involves a volunteer convenience sample that may not fully generalize to the broader population. While the inclusion of an adult lifespan sample has significant advantages, as reviewed above, there are systematic changes in the factors under study that are known to occur with age. Careful consideration of how to bring chronological age into analytical models will need to be given (eg, as a continuous factor, through group stratification), as well as attention to how changes in the effective sample size within each strata (younger/older, decade by decade) affect statistical power. Finally, while EMA studies that examine how individuals behave in response to everyday exposures can be viewed in many ways as naturalistic experiments, the project still involves an observational study design, and findings will require further experimental work to tease apart causality and mechanism of action.

### Conclusions

The PHIAT project involved a novel study design and an adult lifespan sample. The design included near-continuous sampling of thoughts, emotions, context, cognition, and behavior, and is amenable to a wide array of multilevel, time series, latent variable, and other modeling approaches for developing insights into these rich and nested data streams. The inclusion of community-dwelling adults ranging from 18 to 89 years of age will help provide insights into how self-regulation of health-promoting behaviors changes throughout the lifespan and help identify potential mechanistic targets for health promotion interventions.

## Abbreviations

EMA: ecological momentary assessment
M2C2: Mobile Monitoring of Cognitive Change
MRI: magnetic resonance imaging
PA: physical activity
PHIAT: Physical Activity, Imaging, and Ambulatory Testing
